# Assessing implementation outcomes for launching balanced energy protein supplementation: A formative study in rural Bangladesh

**DOI:** 10.1111/mcn.13606

**Published:** 2023-12-13

**Authors:** Anna Kalbarczyk, Mary de Boer, Nazrana Khaled, Barnali Chakraborty, Atiya Rahman, Eleonor Zavala, Hafizur Rahman, Hasmot Ali, Rezwanul Haque, Kaniz Ayesha, Towfida J. Siddiqua, Kaosar Afsana, Parul Christian, Andrew Thorne‐Lyman

**Affiliations:** ^1^ Department of International Health, Bloomberg School of Public Health Johns Hopkins University Baltimore Maryland USA; ^2^ James P. Grant BRAC University Dhaka Bangladesh; ^3^ The JiVitA Project Johns Hopkins Bloomberg School of Public Health Rangpur Bangladesh

**Keywords:** ANC, Bangladesh, implementation, nutrition, supplement

## Abstract

Balanced energy protein (BEP) supplementation is an efficacious intervention in pregnancy for improving birthweight and is recommended by World Health Organization (WHO) in countries with high maternal undernutrition. Few countries have implemented BEP programmes due in part to high cost, lack of data on acceptability and feasibility, and complexity of delivery. We sought to address implementation gaps in BEP interventions through a formative study designed to understand implementation outcomes. We conducted 52 in‐depth interviews and 8 focus‐group discussions with married women of reproductive age, family members, health care providers and pharmacists in three unions of the Gaibandha district in rural Bangladesh. Interviews were translated and transcribed in English and analysed using an analytic framework for implementation science in nutrition. BEP was viewed as an acceptable and appropriate intervention to combat undernutrition in this setting. There was a lack of clarity on who should or could be responsible for providing/distributing BEP in a way convenient to mothers. Many participants preferred door‐to‐door delivery and thought this approach could address social and gender inequities, but providers mentioned already being overworked and worried about adding new tasks. Participants were concerned about the affordability of BEP and opportunity costs associated with travel to proposed distribution sites such as ANC or pharmacies. Women in these communities do not always have the agency to travel without supervision or make purchasing decisions. BEP supplementation is a complex intervention; future trials seek to assess ways to overcome these implementation challenges and inform a long‐term systems‐owned BEP intervention.

## Key messages

- Balanced energy protein (BEP) supplementation is an efficacious intervention in pregnancy for improving birthweight but its implementation through ANC, as recommended by WHO, is complex.
- Decisions about who bears the cost of the product should consider the feasibility of scaling solutions to different groups. Targeting the most in need and with government supported programmes should be considered.
- Door‐to‐door distribution emerged as the preferred delivery modality, though the feasibility of this mechanism at scale is a concern, particularly given the documented lack of human resources in health.
- Given the positive community perceptions around BEP supplements, delivery through ANC could increase ANC participation.

## Graphical abstract

While balanced energy protein supplementation is seen as an acceptable intervention by women, their families and health care providers, its implementation and rollout will be complex. Who bears the cost, who delivers the product and how it is delivered are important concerns which we will seek to address.

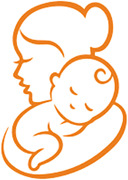

## 1 INTRODUCTION

Undernutrition is a pressing and pervasive health problem for pregnant women and women of reproductive age, particularly in low‐income and food‐insecure settings (Heidkamp et al., 2021; Victora et al., 2021). The recent Lancet Series on Maternal and Child Undernutrition highlighted the need for more implementation research (IR) to increase coverage of current interventions and to expand the implementation of interventions with proven efficacy (Heidkamp et al., 2021). Many of the maternal nutrition‐focused interventions recommended in this series and World Health Organization (WHO) guidelines, such as the delivery of iron folic acid, calcium, and multiple micronutrient supplements (MMS) are most often delivered through antenatal care (ANC) services (World Health Organization, 2016). However, such interventions often suffer from low coverage and adherence, the result of low use of ANC, supply chain bottlenecks, and other challenges.

Extensive research from randomized trials suggests that balanced energy protein (BEP) supplementation in pregnancy is effective in improving birthweight, particularly in undernourished populations (Lassi et al., 2021). BEP supplementation in pregnancy is recommended by WHO for settings in which the prevalence of maternal underweight exceeds 20% (World Health Organization, 2016). Few countries meet this threshold at a national level (Christian et al., 2020). Increasingly high rates of undernutrition are found at subnational levels, suggesting that targeted approaches to address nutritional deficiencies within countries could be an opportunity, particularly given widening inequities in nutritional status in many countries (Victora et al., 2021).

While evidence of the efficacy of BEP has existed for more than two decades (Kramer & Kakuma, 2003), few countries have implemented BEP supplementation programmes outside of humanitarian emergencies. This to a large extent is due to the perceived high cost of implementing food supplementation programmes, although other challenges include a lack of data on the acceptability and feasibility of implementing programmes and evidence of successful delivery modalities (Das et al., 2018; Imdad & Bhutta, 2012; Ota et al., 2015). While there are some experiences implementing food supplementation programmes as part of or alongside health care, such as food supplementation along with antiretroviral treatment for HIV/AIDS, food commodities have many features that are different from other commodities delivered through health systems. Food products are more complex to store and deliver and may attract pests and therefore pose challenges to storage within health facilities (Endris et al., 2023). In food insecure communities, food may be in high demand, and efforts to target it towards some beneficiaries may not be well understood by communities and can introduce challenges. There are also increased risks of sharing, particularly in contexts of food insecurity (de Kok et al., 2021), reducing possible benefits to the intended recipient (Harris‐Fry et al., 2017).

IR offers frameworks, theories and methodologies designed to support decision making for implementation or de‐implementation of interventions. Many calls have been made for IR in the nutrition space and to better understand the acceptability and feasibility of implementing BEP supplements (Kurpad & Sachdev, 2022; Shekar et al., 2021). To some extent, IR has been used to understand barriers and facilitators to the implementation of certain nutrition interventions (Athavale et al., 2020; Endris et al., 2023; Martin et al., 2018), but the necessity of expanding the application of IR in the field of nutrition has been widely acknowledged (Garrett, 2008; Heidkamp et al., 2021; Menon et al., 2014; Tumilowicz et al., 2019).

The aim of this paper was to address implementation gaps in the BEP intervention space and explore implementation outcomes and different distribution modalities for BEP supplementation in pregnancy in an undernourished setting. The implementation outcomes of interest included acceptability, adoption, appropriateness, costs and feasibility; we did not explore outcomes generally associated with later stages of research such as fidelity, penetration and sustainability.

## 2 METHODS

We conducted a qualitative formative study to assess key implementation outcomes related to BEP supplementation among pregnant women in Gaibandha, Bangladesh, located in the rural northwest and as part of the study area of a maternal and child health and nutrition project called ‘JiVitA’. The context is described extensively elsewhere (Labrique et al., 2011). Implementation outcomes are distinct from service and treatment outcomes; they are ‘the effects of deliberate and purposive actions to implement new treatments, practices and services’ (Proctor et al., 2011).

The research included in‐depth interviews (IDIs) and focus‐group discussions (FGDs) with married women of reproductive age (WRA), family members, health care providers (HCPs) and pharmacists. The study was conducted in three unions of the Gaibandha district—Bamandanga, Sonarai and Sarbananda—largely rural areas reliant on an agricultural economy and with some of the highest poverty rates and lowest socioeconomic development indicators in Bangladesh (data.bbs.gov.bd/index.php/catalog/182).

We applied a criteria‐based selection technique, working closely with our local female community health research workers (CHRWs) who reside in the community and were hired for and have enrolled women in previous JiVitA studies. CHRWs administered a general recruitment script to provide participants with information about the study and administered screening questions to collect information on individual characteristics. With this data, sampling frames were developed to include a diverse group of women in terms of age, parity and socioeconomic status categories and included women who were currently pregnant and/or had a recent birth. This allowed us to get the most variability in terms of women's perceptions and experience across the three geographical areas.

IDI and FGD guides were developed to explore attitudes and perceptions towards food consumption and supplement use in pregnancy, and to assess the acceptability and feasibility of BEP supplementation implementation in this setting. The semistructured interview guides were tailored to the participant type and captured questions in the following domains: pregnancy health and antenatal care; food and nutrition in pregnancy; BEP acceptability and distribution; information sources; and community and structural support. The food product was also shown to some participants who were asked to reflect on its texture, smell and taste.

Data were collected by three trained JiVitA interviewers. Training took place over a 2‐week period and included qualitative research techniques, field guide pre‐testing and human subjects research ethics. Data collectors were trained to develop ‘expanded notes’ after each IDI/FGD, which involved identification of quotes related to the study aims and direct transcription from the audio recordings. Data collection took place from January to March 2022, followed by verbatim transcription and translation of the expanded notes. Data were stored on encrypted servers and deidentified before analysis. This study received ethical approval from the BSPH (FWA:00000287) and JPGSPH (FWA:00029561) Institutional Review Boards.

### 2.1 Data analysis

**Figure 1:**
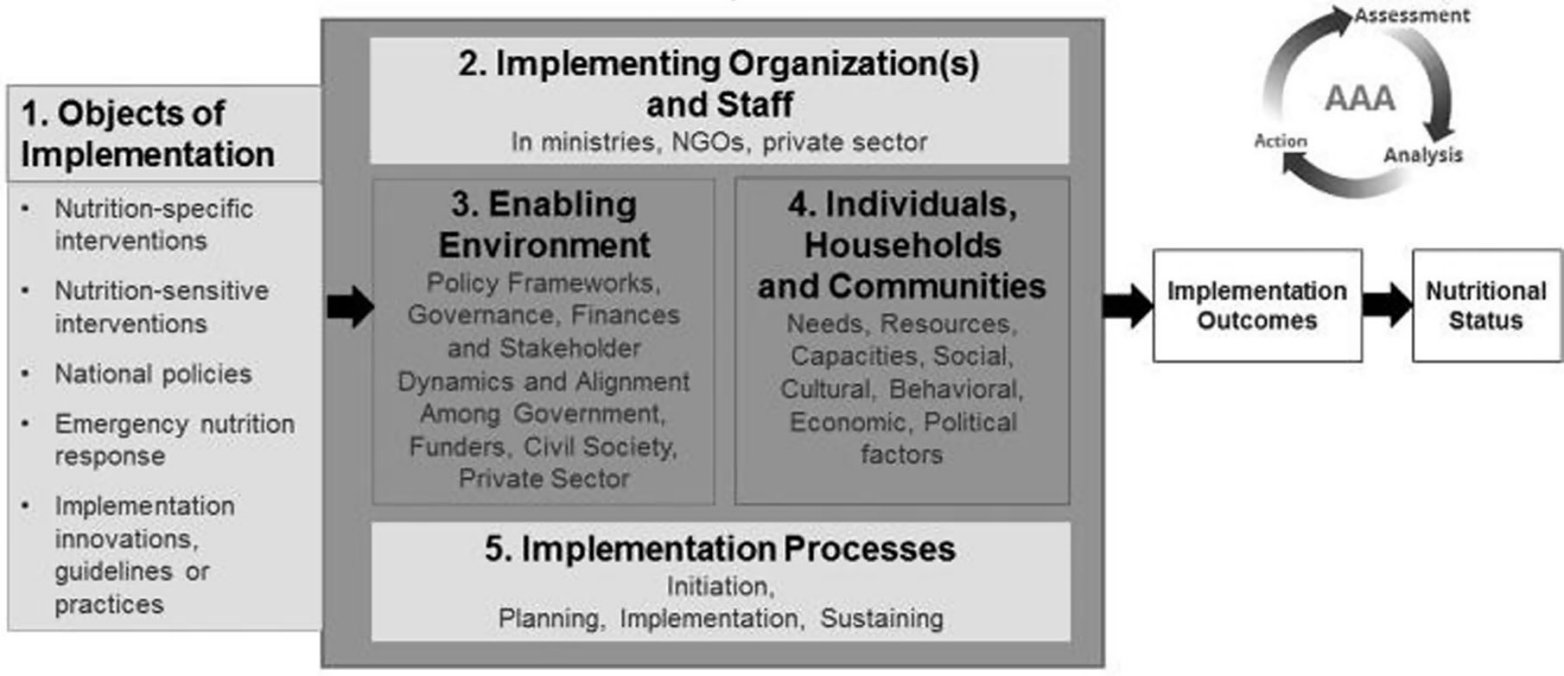
Analytic framework for implementation science in nutrition.

We developed a code list based on a guiding analytic framework developed for implementation science in nutrition (see Figure 1) (Tumilowicz et al., 2019). Codes were generated for each of the five domains presented in the framework in addition to the implementation and nutrition outcomes of interest. For example, in Domain 1 we generated the codes ‘BEP’, ‘ANC’ and ‘Targeting’ to capture specific objects of implementation. In Domain 4, we separated individuals, households and communities into distinct coding categories to explore the nuanced needs, resources and capacities of each group.

Six team members, three from JHU and three from BRAC University, coded the transcripts. After 10% of transcripts were coded, we refined the code list to capture emerging themes and identify relationships. We calculated inter‐rater reliability and used scores to guide team discussions and reorient coding towards common understanding. Data were managed and coded using Dedoose software (version 9.0.62).

While we coded all outcomes, some are more relevant to formative exploratory work than others (Proctor et al., 2011). For example, acceptability and feasibility are highly relevant but without actual implementation, participants are unable to deeply discuss fidelity, penetration or sustainability (generally more salient outcomes in later stages of implementation).

We reviewed analytic charts to assess where BEP, as an object of implementation, was co‐coded with each implementation outcome. We also reviewed codes associated with the implementation process and implementing environment.

## 3 RESULTS

We conducted 55 IDIs with WRA (*n* = 23), husbands (*n* = 6), mothers‐in‐law (MIL) (*n* = 6), health providers (*n* = 9), and pharmacists of the Social Marketing Company (SMC) (*n* = 5). The SMC is a privately run social enterprise that operates pharmacies and health centres in all 64 districts of Bangladesh; the company markets an array of family planning, nutrition, and maternal and child health products at low cost.

We also conducted 10 FGDs with WRA (*n* = 5) and health providers (*n* = 3). In total, we recruited 95 participants; the breakdown is presented in Table 1.

**Table 1:** Participants in IDIs and FGDs.

Participant type	Data collection method	Number of IDIs/FGDs	Total number of participants
Women of reproductive age	In‐depth interviews (IDI)	23	23
	Focus group discussions (FGD)	5	29
Husbands	IDI	6	6
Mothers‐in‐law	IDI	6	6
Health providers	IDI	9	9
	FGD	3	17
Social Marketing Co (SMC)	IDI	5	5
Total		60	95

Abbreviations:  IDI, in‐depth interviews; FGD,  focus‐group discussion.

We initially organized our findings by early implementation outcomes, including acceptability, adoption, appropriateness, costs and feasibility. Key results and illustrative quotes, mapped to each outcome (and its operational definition), are presented in Table 2.

**Table 2:** Findings and Illustrative quotes by implementation outcome.

Implementation outcome and definition	Findings	Key quotes
*Acceptability* (the perception among stakeholders that a given treatment, service, practice or innovation is agreeable, palatable or satisfactory; can encompass content, complexity, comfort, delivery, credibility, etc.)	– Traditional concerns regarding limiting gestational weight gain persist and may pose an obstacle to consuming BEP – Families trust JiVitA to offer them good products and services. – The best way to deliver BEP is through local, door‐to‐door distribution; this allows the product to be given directly to the beneficiary and to conduct health promotion/answer client questions – Health workers have limited capacity to take on additional responsibilities – Clinics were seen as less acceptable sites due to distance/travel time, corruption, and HCP workload – BEP was viewed as broadly similar to existing products—this increases its acceptability but may also limit its necessity	– HCP: There are still some superstitions in our community, there are some wrong concepts among people that if the pregnant women take supplements or iron tablets then the baby will be larger. Then she can't give birth to the baby without a cesarean section, there are still some rare areas like that. (HCP, IDI‐036) Re: Trust in JiVitA – Husband: As different NGOs provide different types of foods and services, ‘they’ (JiVitA) will never give us bad things. If they want to give those products for money, they can do that, but they will give us good quality things. Price is always a price, whether it is one taka or ten taka. (Husband, IDI‐1098462) RE: Local delivery – HCP: The advantage of giving products [at home] is that the mother is able to get it directly in her hands; she can directly get it and start eating it. And it would be possible to explain all the things to her in person. (HCP, FGD‐01) RE: HCP overload – HCP: We can't take this trouble. We already want to quit this job. There is too much workload! (HCP, FGD‐03) RE: Clinics – HCP: If you distribute ‘it’ at the clinic, how many mothers will take this service from there? And how many mothers go to the health clinic? It's not like they don't go, but the number is much less. (HCP, FGD‐01) RE: Similarities to existing products – MWRA: I ate a packet of Mother Horlicks. Eating Mother Horlicks will nourish mother and baby. I would have eaten Pregvita if I had known about this type of packet; if I had known about Pregvita, I would have eaten it. (MWRA, IDI‐655877)
*Adoption* (intention, initial decision, or action to try to employ an innovation; Uptake, utilization, intention)	– To address concerns, such as eating down, BEP would have to be promoted within the community—both through HCPs and among women themselves – Mobile phones are one possible promotional method, but many women have limited/regulated access to them	RE: Necessity of promotion – MWRA: BEP will definitely be good for people. But first you have to promote it. For example, I am eating it today, I will obviously tell this to the people at home. I will tell them that I got so and so type of food. If I don't tell, and if you don't show, then nobody will understand what this is. (MWRA, FGD‐02) RE: Power of promotion – HCP: If we sell this in the villages, if we make the pregnant mother understand that she has a nutrient deficiency, and that if you eat this packet, then 23 micronutrients will be consumed in your body, the deficiency will be met up, that this should be eaten in this way—the way we will preach, the pregnant mother will obey (HCP, FGD‐02) RE: Mobile phones – MWRA: I don't have any personal phone. My husband does not let me operate his phone, he does not let me touch it. If I need any information, I need to tell my mother in law, then she may allow me to use her phone. (MWRA, IDI‐852261)
*Appropriateness* (perceived fit, relevance, or compatibility for a given setting; Usefulness, practicability)	– People in this setting are poor so BEP can help with nutrition. – Pharmacists emerged as potentially powerful product promoters. – Pharmacists work on commission and may already be selling competing products	RE: Utility – MWRA: Women need to be told its nutritional value, so it can be suggested to their family. During pregnancy, women have some doubts on what they should eat; what if there are any problems? (MWRA, FGD‐02) RE: Pharmacists as promoters – SMC: What are the benefits of eating [BEP]? We will say that it will be nutritious, the growth of the baby will be good, your body will be able to prevent anemia, you will have good energy in the body, it will keep your body fresh and you will be free from weakness. (SMC, IDI‐048) RE: Competition/compatibility with existing products – SMC: I am also working as a SMC provider and giving Fullcare, so they may think negatively about me providing Pregavita. If Fullcare works the same as Pregavita does, then there is no need of keeping Pregavita here. (SMC, IDI‐005)
*Costs* (cost of an impact of implementation effort)	– Household access to financial resources are deeply connected to feasibility. – Differing opinions if BEP should be given for free (has implications on design and success of future programs) – Families are aware that they need to supplement pregnant women, but cannot afford the supplements currently available – The local population is very price sensitive … – … and the most price sensitive are those who are most vulnerable to malnutrition and most in need of BEP – Price will determine how much stock a women will be able to take at once – Taking smaller quantities at one time may lead to lower adherence/greater risks of supply interruption – Other NGOs who charge for similar nutritional supplements/products have had difficulty with uptake (of those products) – Women have little financial independence—their access to resources is mediated by their husbands and their mothers‐in‐law	RE: Knowledge but no ability – Husband: She does not take vitamin and iron tablets. Brother, there is a reason, they have money, I am talking about rich people. Poor people like us will not eat those, we are afraid, we do not have money; you have to understand. Rich people will feed them (pregnant women in their families) a lot. The food that we can't eat because we can't digest it or don't have the taste for it, the rich will feed their families these things. They have to bring it from wherever they can. With the little amount of money I earn, if my wife wants to eat something, I bring it for her and feed her. I bring it myself and feed her. But it is not possible for me to bring it regularly. (HUSBAND, IDI‐1034988) RE: Price‐sensitivity – MWRA: If you ask us to take one per day, in my opinion, then the middle and lower class or poorer people cannot eat regularly. I don't think they will be able to eat this regularly if the price is more than 10 ($0.10 USD) to 15 ($0.15 USD) taka per packet. (MWRA, FGD ‐02) RE: Price‐sensitivity related to neediness – SMC: How much should the price of Pregavita be if it is being sold? Now, these are being consumed by the poor people, who have nutritional deficiencies. The need is more for the poor people. This is not consumed by anyone other than the poor people. The rich people don't take these foods very often. Most of the time the foods they take don't need this nutritious food packet. But the pregnant women who are very poor and from their level whatever they can afford, they can afford only a small amount of money. If you sell it at a low price, then the poor people will be able to benefit. They will be able to buy the packet if the price is 5 ($0.05 USD) or 10 taka ($0.10 USD). If the price limit is within 5 to 10 taka, then 80 percent of people will be able to buy the BEP. If the packet costs 5 taka then they will be able to buy 20 packets at a time or 10 packets at a time. There may be no problem. They will eat 10 food packets in 10 days. And whenever they will finish the packets, they will again go and buy 10 more packets, and then they will eat them. You know, they will not be able to come every day to take one packet. (SMC, IDI‐005) RE: Effect of cost on supply – HCP: There are also such mothers who will take 30 packets at a time in their vehicle, and there are also such mothers who do not have the ability to buy 30 packets at a time, her husband works as a day laborer. The day laborer's wife can take 5 packets, then when they manage more money, she will take 5 more packets. But they will eat sacks of these if they are given for free. (HCP, FGD‐02) RE: Effect of cost on adherence – SMC: For the frequency of intake, now most of the people in the village are poor, they can't maintain the full dose, Either due to need or due to negligence, actually the thing that I try to make them understand that she has to take medicines for certain days to remain healthy and to keep the baby healthy, but they have not that much amount of money for that reason they don't complete the full dose. If I say to them to take the medicine for a certain amount of time like 3 months or 6 months, most of them don't complete the full dose or I am not sure they may buy the medicine from another shop. In this situation they take treatment, the number of the patients taking the full treatment or full service are very low. Actually they don't want to complete the full dose. (SMC, IDI‐049) RE: Experiences of other NGOs – HCP: For example, no one wants to take the things given by BRAC…We have a BRAC health worker over at my area. She takes 20 taka (~$0.20 USD) for a strip of calcium, [women and families] don't want to pay that 20 taka. That woman sometimes brings back the medicines because no one takes them. And since she cannot sell, she takes it herself and gives the money out of her own pocket. It's like that. (HCP, FGD‐03) RE: Women financially dependent on men – HCP: The women of our country are actually financially completely dependent on their husbands. It is seen that they don't have the ability to give the support for coming to the hospital by rickshaw, auto or van. Such low income people are also residing in our society. In terms of cost, of course it can be an obstacle. (HCP, IDI‐414) RE: Women financially dependent on in‐laws – MWRA: Family does not cooperate to get health services. They tell me not to take healthcare. Now I am pregnant, but we have less money. There are many mothers‐in ‐law who say insulting things (*gali dey*), they don't want to spend any money. I want to go to my father's house for treatment…They say, there is no need (to go to your father's house); it is our son, we will manage. If there are any problems, we will admit you in a clinic; or if the delivery can happen at home, then that will be ok. They will be the ones to take care of these things, my in‐laws. They won't let me go home for the cost. (MWRA, IDI‐852261)
*Feasibility* (extent to which a new innovation can be successfully used or carried out within a given setting)	– Clinicians shared they had limited bandwidth to conduct additional ANC promotion within their crowded work days – Satellite clinics were seen as inconvenient for both the women attending them and the providers operating them – Transportation was one of the biggest barriers for accessing BEP at either clinic or satellite sites – Field workers emphasized their previous training to deliver similar products door‐to‐door and beliefs that BEP could be distributed similarly – Women emphasized that only a moderate quantity of BEP should be supplied at any one time to prevent sharing/wastage – Voucher systems were seen as feasible but only if the reporting burden on pharmacists was low	RE: Limited bandwidth at clinics – HCP: After providing services, I have to do other work, too. So, it is difficult for me to find time to talk to them. (HCP, IDI‐015) RE: General undesirability of satellite clinics – HCP: If they come here with time on their hands, then there is no problem. We give services there over the entire day, but if something is a little amiss then there is a problem. From my end, I can tell you that it is an inconvenience. It is difficult for me to commute to and from certain places which don't have proper transport access. (HCP, IDI‐101) RE: Transport barriers and importance of local delivery – HCP: Yes, it will be a problem for them if they have to go to pick the BEP up from somewhere. As a poor person, they may not have the financial ability to go there. If they can't get the service from us, then how would they be able to spend 10 taka and go there? Isn't that a bit weird? That's why it would be good to give it at the field level. (HCP, FGD‐01) RE: Suitability/training of field workers for this role – HCP: We work at BRAC. As we have some trainings from BRAC, we have some idea as BRAC gives us Iron, Calcium to distribute. They have definitely given us training on that. We can make them understand what ingredients are in it, and how we will distribute it when you give it to us. (HCP, FGD‐01) RE: Quantities at one time – MWRA: It would not be wise to give it all at once. It will be too much all at once. It will be better if you give the quantity for 1 month or 15 days. When you give 180 packets at a time, it's a huge amount. Then we will think, in how many days would we be able to consume all these packets? When you have 180 packets, then if anyone wants one packet from you, that time you might think that there will be no problem if I give her one packet (out of so many). Many will think like this. In this way, there may be a waste of some packages. And when you give a smaller number of packets for 14 or 15 days or for even for 1 month, it will be more suitable. Then we can say that we must eat it for 1 month, we can't share it. Then we can eat it alone. Other people will understand also. And if you give too much at once, then they will say, you have a lot, there will be no problem if you give one. (MWRA, FGD‐TIPS‐FU‐01) RE: Voucher systems need to be simple – SMC: Here is the problem in keeping accounts … Such as, if they come with the voucher and I give them the product, this is alright. But when I will give them the product, and I have to write down the calculations in a notebook like SMC … We face a lot of problems in doing this. We face a problem of deciding whether we will consult with the patient or keep track of the calculations. If it involves writing down the calculations then I will not be able to do this. (SMC, IDI‐005)

BEP was widely viewed as *acceptable* though some were concerned about its value‐add given existing products (described in detail below). However, participants noted that people in this setting are poor, so BEP is an *appropriate* intervention for addressing undernutrition in pregnant women. Household access to financial resources and the *cost* of BEP were important considerations, though participants differed in their opinions about whether BEP should be distributed for free. Participants agreed that promotion would be critical to influencing *adoption* of BEP, and pharmacy owners were viewed as potentially powerful promoters. The *feasibility* of BEP as an intervention depended on delivery modalities and payment systems (i.e., subsidy vouchers or direct payments).

Across implementation outcomes, three overarching themes emerged, (1) the role of BEP in combating undernutrition, (2) responsibility for BEP distribution and (3) implementation and product costs. Each theme is described in further detail below.

### 3.1 Appropriateness of BEP and need for promotion

Participants raised some concerns about the appropriateness of adding BEP to the existing arsenal of tools used to combat undernutrition. Community members—including women, their mothers‐in‐law and their husbands—as well as HCPs described other products that they are already aware of such as Mother Horlicks (a fortified, high‐protein drink mix produced by Unilever‐India and marketed to pregnant women) and Fullcare (antenatal multiple micronutrient tablets, recently piloted as part of the local SMC network), and some questioned the added value of BEP. One HCP reflected that since these products are already well accepted, women may not understand or quickly accept BEP.

Mothers will see this (BEP product) and say, what is this, baba?! This is a new thing; (I don't know) what will happen if I eat it or not! But since Mother Horlicks is widely accepted, it is being sold in the markets and mothers are eating it, then it will be easier to make mothers understand (if it is in a similar form as Mother Horlicks). But it will take some time to make mothers understand.  (HCP, FGD‐01)

Promotion of the product was viewed as key to increasing its acceptability and combating beliefs among some in the community that not consuming additional food in pregnancy might or to use supplements facilitate an easier birth and a smaller infant. While few women expressed this belief themselves, multiple health workers recalled that women had raised such concerns with them previously in relation to calcium or iron supplements. As one health provider shared:

I hear from many mothers in the village that if they eat iron and sulphate supplements, the child will grow large, and the mothers are bothered by this. We give them these supplements along with the check‐up, but they do not want to eat them. They think that it will make the baby grow a lot, that it may interfere with the delivery of the baby. (HCP, IDI‐100)

Addressing these concerns will be key to ensuring uptake, however—as women themselves underscored, perceptions of delivery complications linked to use of food products is just one of many questions that women may have regarding a new product:

Many will ask: what will happen with this food packet that you are giving? Why did you bring it? Will there be any problem if you eat this? Since many people don't know about it, they may have some hesitations regarding it. You may need to break down the answers to the questions if necessary. You may need to say: eating this food will keep you and your baby healthy, physically fit, and your unborn baby will be well. (WRA, FGD ‐ TIPS 01)

Another woman explained that such hesitancy regarding a new product, consumed during pregnancy, was only natural, further emphasizing that good communication and promotion approaches would be crucial:

No one wants to take a risk during pregnancy. They have doubts in their mind about what to eat, what will happen to the baby. They will wait and see what happens if this is eaten. You have to make them understand, especially initially, you have to make everyone understand. (WRA, FGD‐02)

Many health workers felt, however, that such questions would be simple to address with good counselling and evidence of good results among women who consumed the product. This was primarily, in their views, because the intentions of the mother and of JiVitA were in accordance. As one health worker recounted:

Mothers want their baby to be well nourished and healthy. Those who do not understand will understand if you explain it to them. (MWRA, FGD‐TIPSFU‐02)

The impact of positive results on precipitating behaviour change was particularly important:

I would say it's like a chain. If a pregnant mother from the village comes to me and gets well with the service, she must go back to their area or neighborhood and say that this madam has given this treatment and she has gotten well. This is the awareness that if a woman in the next house becomes pregnant, she thinks that “let's go to that madam and take the service.” This is a good thing that this is running like a chain. (HCP, IDI‐414)

Overall it was felt that interest and willingness to adopt the new product existed, but promotion—including patient, direct communication from providers to their female clients and their clients’ families as well as between and among clients themselves—would be necessary to establish trust in the new product.

One type of promotion that interviewers investigated was the use of mobile messaging, with the aim of reminding women to consume the product daily as well as to pick up new supplies of BEP when their existing stock was exhausted.

Opinions on the appropriateness of mobile messages for this type of communication varied. Women shared that mobile messages were not currently in use for distribution of any other type of pregnancy‐related information, but appeared open to receiving communication this way, saying:

*A*bsolutely, if anyone sends me messages or calls me to give me pregnancy‐related information, then I will abide by this advice. (WRA, IDI‐272149)

And,

That's also great if they remind me using SMS. I will have more interest in eating the product, then. (WRA, FGD‐TIPS‐03)

However, despite the professed ubiquity of mobile phones in Bangladesh, many women shared that the mobile phones in their household belonged to the men or to their mothers‐in‐law. As a result, the timeliness of communications and/or the ability to actually receive the intended communication was called into question. As one woman recounted:

Suppose, the mobile may be with someone or maybe their husband keeps the mobile with him, or their son may keep it with him. They go to work at different places. Suppose you send us a message, but at that time they are driving. When they come back home, it's too late and they forget to tell me about the message. Suppose you advise me to eat at 3 pm, but if he comes back late at night, then that will be a problem.

In other households, where the mother‐in‐law was the mobile phone owner, this put the mother‐in‐law in the role of information‐gatekeeper.

### 3.2 Responsibility for BEP provision

While interviewees agreed that it would be important to provide BEP in a way that was convenient and acceptable to pregnant mothers, there was a broad lack of clarity on who should or could take on this responsibility.

#### 3.2.1 Door‐to‐door distribution

Among health care providers, there was general consensus that distribution at the field level was the most effective means of ensuring uptake. Such an approach was seen both as highly feasible and acceptable, with the possibility to build on existing programmes, for example, BRAC, where community health cadres conduct monthly checkups on pregnant women and provide them with needed medications.

Health care providers emphasized that, in addition, door‐to‐door distribution would minimize travel times and costs by mothers. Several noted that distance and time were crucial de‐motivating factors for women, observing:

If it's far, they cannot go and don't want to go. (HCP, FGD‐01)

Door‐to‐door distribution would also ensure that the packets were distributed to the women who needed them most, and that any explanations of how to use the product and/or any concerns that the mothers or their family members had about consuming BEP could be addressed directly by the community health workers or the health providers themselves, as these visits would be less harried than those at a formal health facility site.

Indeed, addressing intra‐household dynamics was highlighted as a particular benefit of door‐to‐door work, and would be crucial to ensuring women would be allowed to access BEP. The ability of both the pregnant woman and her family members, particularly her mother‐in‐law, to ask a provider questions during such household visits was highlighted by many women interviewees. As one woman emphasized:

When the husband brings it, the mother‐in‐law will think of something else; then they will say why are they buying something that costs this much money? There is a lack of this and that in the family. I cannot pay back the loans, but look what they are bringing from the store. They will surely say such things. But when you go door to door, the mothers‐in‐law will see you, then you can explain to everyone, then they will understand that it is actually a good thing. (WRA, FGD‐TIPSFU‐02)

The principal barrier to door‐to‐door distribution, however, was provider workload. While several facility‐based HCPs noted that doing field work was an essential part of their responsibilities, they also underscored their substantial existing responsibilities. Important concerns were raised as to whether BEP distribution could reasonably be added to this already large workload. According to one provider:

If you force the work upon us, we cannot just deny it, but we cannot do it either. It is better not to give us this responsibility. (HCP, FGD‐03)

One provider added that the only way to ensure they would be able to pay attention to an additional service like BEP was to offer incentive payments:

There definitely needs to be a service charge if you want our workers to give their 100 percent for this. You have to provide a service charge to those doing the distribution. Nobody will work without this. (HCP, FGD‐01)

Such bandwidth limitations raise important concerns about the feasibility of adding BEP to either health care provider's field‐work responsibilities or to their clinical roles.

#### 3.2.2 Distribution through ANC

Concerns about the acceptability of adding BEP to ANC clinical interventions were further highlighted by women themselves. While clinics were recognized as a feasible site—women noted that a community clinic exists in every ward and that women are accustomed to attending them for specific services on fixed, pre‐advertised dates—many also emphasized that the crowded, busy clinics do not provide high quality services. As one woman recounted,

Some pregnant women don't want to go to clinics at all because they don't even see you properly. I went there the other day to get vaccinated, and they just gave me the vaccine but didn't behave well to me. They shooed me away. Let alone measure my weight! They didn't say anything. (WRA, FGD‐TIPS‐03)

Clinics were also seen as an undesirable location due to perceived corruption and diversion of medicines. As another interviewee shared,

If you give these packets at the clinic for 5 taka (~$0.05 USD), or 10 taka (~$0.10 USD), the clinic will charge 5 taka (~$0.05 USD) more. They will sell it elsewhere, women will not get it. They don't give the general medicines to the public. The government supplies the medicine to the hospitals in the hope that they are working hard to save the lives of the general people. To take care of the sick children. But when you go to providers at the hospital, they say that there is no medicine. If you give it to one person, you fill one person's stomach, if you give it to the clinic, not everyone gets it. (WRA, FGD‐TIPS‐01)

Finally, concerns were raised about access barriers when distribution of products was conducted at more distant, less friendly sites, with both providers and women echoing one another in saying that distance would (and does) affect the numbers who access care and receive formal ANC counselling.

However, HCPs reflected that it was easier to provide information when women come directly to them at the clinic.

#### 3.2.3 Pharmacies

Local pharmacies were viewed as convenient and acceptable places for accessing BEP both by women and their husbands, especially given the large SMC pharmacy network that exists in Bangladesh. As one woman recounted,

Everyone goes to the market all the time, even if the women don't go, the males always go to the market, they will be able to collect BEP there [with] no problems. (WRA, FGD‐TIPS‐03)

Moreover, the role of the pharmacy shopkeeper as a promoter of products was emphasized by both women and pharmacists alike. In the words of one woman:

When they go to buy medicine, the shopkeeper will say I have a very nice nutritious product, you can take it. Then many people will be able to take it. (WRA, FGD‐TIPS‐02)

The similarities between BEP and existing products sold in pharmacies was also mentioned—most notably the Monimix micronutrient powder for children under 5 years old, which is distributed through multiple channels, including both pharmacies and door‐to‐door.

This similarity in products, however, also led the pharmacists to voice concerns about competition between BEP and existing socially marketed products. Pharmacists mentioned that they were newly under contract to sell a multiple micronutrient supplement for pregnant women called FullCare, and many were concerned that they could lose their commission if they offered a competing supplement. As one pharmacist observed,

If my [Social Marketing Company (SMC)] tells me that I can keep the Pregavita as well as the FullCare, then there may not be any problems. But if they are adamant that if there is an SMC product, then it is better not to sell any other products, in that case my commission may be canceled. (SMC, IDI‐005)

All pharmacists were interested in protecting their existing SMC commissions, money earned through selling existing products.

One route of offering BEP in pharmacies would be to subsidize and incentivize the sales via vouchers. Like mobile messaging, women shared that they were unfamiliar with voucher systems but appeared open to them. By contrast, SMC pharmacists interviewed were familiar with voucher‐type programmes and found them onerous. Concerns centred on the troublesome need to make calculations and keep track of vouchers, by the pharmacist, and the risk, by women, of losing the vouchers or tokens and expecting services, nonetheless.

Overwhelmingly, the pharmacists emphasized how busy they were with patient consultations as the reason for considering voucher‐type programmes as less tenable. These regular patient interactions—which make the pharmacists ideal product promoters—could render additional administrative demands of BEP sales distracting and, at times, unfeasible. Most pharmacists stated that they would not be interested in offering the product due to this additional bookkeeping burden. In the words of one pharmacist,

If it is necessary to let you know every month how many packets went out, that means a calculation that we have to do, then it will not be possible. As I already have trouble to deal with, moreover if I take your thing that should be calculated and reported to you, this will be a large trouble for me. If I have to write things then it will be a trouble for me. If it involves writing down the calculations then I will not be able to do this. (SMC, IDI‐005)

Several solutions were proffered, including encouraging women to have a register book or simply keeping a box in the pharmacy where tokens could be deposited and handed over to the JiVitA team without necessitating calculations.

### 3.3 Financial and opportunity costs

Participants were concerned with the monetary cost of the BEP. Some believed that BEP needed to be distributed widely and for free, while others, including WRA and HCPs, felt that women who need the BEP will purchase it. Participants also cited other health and nutrition programmes that provided services for free; unlike with participants in these programs, government providers are unable to back up their health and nutrition recommendations with direct support, which frustrates them. As one HCP remarked:

When I advise them to eat nutritious foods, at that time many say ‘why do you not buy those and give it to us? How will we manage so many types of food? Since you advise us to eat nutritious food, then why don't you provide those? You give us nutritious food. You are doing a job, you can give [it to] us'. (HCP, IDI)

Agency was linked to financial cost as some women reported being unable to make purchases without their male partners or other men in the household. One MWRA described the role of male family members who generally helped her purchase necessary health goods.

I take iron, calcium, and vitamin by purchasing [them]. In this month until now, I haven't purchased [them]…because there is no person who can help me to purchase. (WRA, IDI)

An HCP made the linkage between a husband's power and a possible need for free services.

There are many husbands who get angry and say that we don't have the ability to buy all of these things. We can provide food as per our own ability. They only ask for free service, if they are given for free then everyone agrees to have. (HCP, IDI)

Opportunity cost also emerged as an important implementation consideration. One participant noted that community members must leave their daily work to go to the clinic, which has a reputation for long wait times.

Many (pregnant women) come, the clinic opens after many days or it opens once every week. Many people go there to take medicine, children go (that is, mothers go to take medicine for their baby), pregnant women go, everyone goes to the same place, so isn't it natural that it will be crowded? … Due to the crowding, many do not go there. The villagers have to leave their daily chores to go to the hospital, they cannot wait too long at the hospital, they don't want to stay long, and they come back home (soon). Yes, (if they go to the hospital) it takes a long time and they can't do any work. It takes too long if they go there, so that is why many people don't go. (HCP)

The perception of cost as a barrier varied across socioeconomic tertiles, although socioeconomic status (SES) data was captured only among MWRA. Among the highest wealth tertile participants, many shared that—in their opinion—cost did not form a significant barrier to accessing antenatal care services, including the purchase of additional medicinal or food supplements in pregnancy. High SES women noted that, in their opinions, those in the lower SES tertiles were more likely to be sensitive to the price. None of the high SES interviewees insisted BEP should be free. Rather, as one interviewee described:

I think they would like the packaged food. It's easy to purchase from the market […]. Nowadays, Mother Horlicks is priced a little high, so many people don't have the ability to purchase it. If the price of the packaged food was less, and if it was easily accessible, then people would like it. Yes, everyone would have it if the price was low. (WRA, IDI‐643759)

Others in the highest wealth tertile appeared relatively blasé in assessing the barrier that cost might pose to accessing antenatal services for those in less affluent households. As one high SES husband observed:

It costs money, depending on the cost, the ones who have less money, they go less. Those who have a lot of money can go several times. (Husband IDI‐1522967)

The same husband went on to link SES with the ability to follow ANC guidelines, noting:

If we don't [follow guidelines], then it may be seen that there are some complications at the time of delivery. Then, you have to take the mother to a clinic or hospital. Then the cost increases rapidly and all of a sudden. I mean the family has to spend a lot of money within a very short time. On the other hand, if I can spend a small amount of money on health services earlier, then the mother and baby are healthy. (Husband IDI‐1522967)

Costs associated with pregnancy care, in this man's view, were ultimately a worthwhile, money‐saving investment—with pregnancy representing a financial impact that could be mitigated in scope.

Conversely, low SES families were divided on the affordability of ANC services. As one woman noted:

There was no barrier for taking health services for me. Neither hospital distance nor the cost of taking services were a problem for me. (WRA, IDI‐406598)

However, costs did pose a barrier for many others, particularly when it required negotiation among competing intra‐familial priorities. One woman recounted how her comparatively low status in her family meant that her antenatal care was not seen as worthwhile investment:

My family does not cooperate to get health services. They tell me not to take healthcare. Now I am pregnant, but we have less money. The mothers‐in‐law say insulting things; they don't want to spend any money. I want to go to my father's house for treatment, my father will pay for that, but my in‐laws prohibit me from going there. If I go, then they will have to give me some money to travel. They say, there is no need (to go to your father's house); it is our son, we will manage. If there are any problems, we will admit you in a clinic; or if the delivery can happen at home, then that will be ok. They will be the ones to take care of these things, my in‐laws. (WRA, IDI‐852261)

The frustration this type of constraint poses was recounted poignantly by one husband, as well, who shared that:

My [pregnant] wife does not take vitamins and iron tablets. Brother, there is a reason: they have money, I am talking about rich people. Poor people like us will not take those supplements; we are afraid, we do not have money—you have to understand. Rich people will feed pregnant women in their families a lot. These nutritious foods and supplements, the rich will feed their families these things. With the little amount of money I earn, if my wife wants to eat something, I bring it for her and feed her. I bring it myself and feed her. But it is not possible for me to bring it regularly. (Husband IDI‐1034988)

It appears that socioeconomic status can influence the perceived affordability of antenatal care, particularly the affordability of additional costs above ANC attendance, such as travel or the purchase of special foods or nutritional supplements, like BEP.

## 4 DISCUSSION

In this paper we examined the perceptions of community members and local HCPs related to the potential acceptability, feasibility and targeting as an approach for delivering BEP supplementation in pregnancy in a rural context in Bangladesh. Women's socioeconomic status was linked to their perceptions of affordability of care and services, including ANC and food supplementation products such as BEP. Lower cost products may be important even for those who are willing and able to purchase them. Stakeholders varied widely in their perceptions of how best to implement targeted BEP distribution and who should be responsible for its implementation and associated costs; door to‐door delivery was the preferred approach but this is likely not feasible for programs at scale, although has been highly successful for delivery of contraceptive methods across Bangladesh (El Arifeen et al., 2013).

Bangladesh is one of a few countries with a history of implementing a large‐scale food supplementation program aimed at malnourished pregnant women. The Bangladesh Integrated Nutrition Project (BINP) started in 1995 and the National Nutrition Program following that sought to reduce child malnutrition through growth monitoring and supplementary feeding to increase pregnancy weight gain. The program's effectiveness received mixed evaluations, and scholars largely point to deficiencies in implementation strategies, including targeting, as reasons for lack of impact (Nahar et al., 2009; White, 2005). Multiple evaluations identified that ineligible children had received supplementary feeding and both leakage (where supplements are fed to people other than the intended target) and substitution (where participants reduced consumption of other food while consuming the food supplements) were evident (White, 2005). Such experiences point to the contribution that research such as ours can play in identifying potential challenges associated with different implementation pathways and to identify strategies to overcome such implementation pitfalls related to targeting and willingness to pay.

One such challenge is the cost of the product. The prototype product used in the study is not in the market and its market‐value is unknown, although such products (providing close to 400 kcal, 14 g of high‐quality protein, calcium, and 18 micronutrients) may cost about 30–50 US cents per daily sachet. Who bears the cost of BEP, whether this is individuals and/or the government, has important implications for program sustainability. In low‐resource settings, women who are most in need of BEP may be unable to afford it, even if they want it and find it acceptable. These tensions between whether health‐goods should be distributed for free or sold in the market is not new and has been featured in debates for other goods such as insecticide treated bed nets (Hoffmann et al., 2009; Khatib et al., 2008), contraceptives (Radovich et al., 2019) and vaccinations (Savulescu, 2021). At the same time, our findings suggest that households in the upper wealth tertile may be willing to purchase such a product. Ultimately, decisions should be made considering the cost‐effectiveness of the product and the overall approach, but also based on the feasibility of scaling solutions to different groups. Targeting the most in need and likely the most under‐resourced and food insecure with government supported programs needs to be considered, with others accessing such a product through social‐marketing strategies.

Some study participants themselves brought up targeted programmes generally focused on poverty and socioeconomic status, to reduce the overall cost of the program and ensure that the most vulnerable are reached. Such targeting tools exist and include indicators to assess wealth such as food security, asset ownership, housing quality, expenditures and employment (Hargreaves et al., 2007; Morris, 2000) or screening for low BMI and mid‐upper arm circumference (Sisay et al., 2021). Any targeting approach must be carefully designed, pre‐tested and implemented with fidelity, particularly given prior experiences of leakage.

There is a misalignment of expectations for a product such as BEP and associated roles and responsibilities including where and how it should be distributed and to whom it should be distributed (i.e., a subset of pregnant women most at risk, or all women in a community). In other words, the distribution mechanisms need to be determined. All provider types who were interviewed raised concerns about their current workloads; a lack of human resources in the health sector has been well documented for Bangladesh and more broadly in the region (Legido‐Quigley et al., 2019; Rawal et al., 2019). While our participants did not discuss burnout explicitly, we recognize that HCP burnout has become an increasingly prevalent and dangerous issue (Dugani et al., 2018; Ghahramani et al., 2021; Moitra et al., 2021). Programs need to account for the added burden on HCPs and consider implementing recruitment and retention strategies including appropriate and timely remuneration.

Door‐to‐door distribution seemed to be the preferred delivery modality for BEP, even if it was less clear who should be responsible for that delivery. The role of community health worker (CHW) service delivery in rural Bangladesh has been widely studied and research shows CHW services can be acceptable, valuable and effective (Baqui et al., 2009; Jahan et al., 2022; Puett et al., 2013). But CHWs are not a monolith—pay structures, education requirements, training and availability of supportive supervision can vary widely. And one recent study found that ‘younger, better educated, and more experienced [CHWs] with positive self‐efficacy were perceived to have performed better than their peers’ in the delivery of a home‐fortification programme (Sarma et al., 2020). In addition to burnout considerations, programmes leveraging existing CHW cadres need to consider the barriers and facilitators to their success (Adams et al., 2020; Sarma et al., 2020).

While participants largely critiqued ANC as a delivery modality, this avenue may still bear some additional consideration. Community perceptions around BEP supplements are largely positive, and the delivery of BEP through ANC could increase ANC participation, a critical goal for the WHO (World Health Organization, 2016). Use of technology in the implementation of BEP distribution and reminders for adherence could be further explored within the ANC platform. The provision of BEP could also make existing ANC counselling on pregnancy in diet more effective, while acknowledging food taboos and restrictions on women's mobility may be important mediating factors.

A strength of our study is that the JiViTA team has a long history of community presence which likely led to greater rapport and trust with participants. Our analysis also triangulated findings across different types of participants to explore the same topics from different perspectives. However, given that the BEP product is not in the market and had not yet been implemented as a program at the time of data collection, it may have been difficult for participants, particularly non‐providers, to conceptualize what such a program might look like or the possible ways that it could be implemented.

## 5 CONCLUSION

BEP supplementation, as recommended by the WHO, as an ANC intervention is complex, and there is limited consensus among community members and HCPs on delivery mechanisms that could be employed, although the ANC platform is an obvious choice. Building on this formative research, the JiVitA study seeks to assess ways to overcome these implementation challenges and inform a long‐term systems‐owned BEP intervention, testing the effectiveness of an untargeted versus a targeted approach for supplementation during pregnancy.

## AUTHOR CONTRIBUTIONS

Anna Kalbarczyk, Andrew Thorne‐Lyman, Eleonor Zavala, Nazrana Khaled, Barnali Chakraborty, Atiya Rahman and Kaosar Afsana conceptualized the manuscript as part of the special issue. Hafizur Rahman, Hasmot Ali, Rezwanul Haque, Kaniz Ayesha, Towfida J. Siddiqua, Nazrana Khaled, Barnali Chakraborty and Atiya Rahman contributed to the data collection. Nazrana Khaled, Barnali Chakraborty, Atiya Rahman, Eleonor Zavala, Mary de Boer, Anna Kalbarczyk and Andrew Thorne‐Lyman conducted data analysis. Anna Kalbarczyk and Mary de Boer led the data synthesis and initial draft manuscript. Kaniz Ayesha, Parul Christian and Andrew Thorne‐Lyman provided substantive edits. All authors have reviewed and approved the final manuscript.

## CONFLICT OF INTEREST STATEMENT

The authors declare no conflict of interest.

## Data Availability

The data that support the findings of this study are available from the corresponding author upon reasonable request.
